# Might the fungus candida albicans a risk factor for autism? A meta-analysis study

**DOI:** 10.4314/ahs.v23i4.25

**Published:** 2023-12

**Authors:** Emrah Gokay Ozgur, Sibel Balci

**Affiliations:** 1 Marmara University, School of Medicine, Department of Biostatistics, Istanbul, Turkey; 2 Kocaeli University, Faculty of Medicine, Department of Biostatistics and Medical Informatics, Kocaeli, Turkey

**Keywords:** Autism spectrum disorder, Candida albicans, Intestinal system

## Abstract

**Objective:**

Due to the high increase in rate of autism, it has gained great importance to determine the etiology of autism spectrum disorder. The purpose of our study was to assess the role of Candida albicans as a risk factor to cause autism behavior.

**Methods:**

We searched Scopus, PubMed, Web of Science and PsycINFO for articles up to December, 2021. The studies involving children diagnosed with autism spectrum disorder were included. Children' outcomes were selected as Candida albicans (positive) and Candida albicans (negative). Odds ratios were reported using fixed-effect and random-effect meta-analysis. The heterogeneity was assessed by the *Chi-square test* and *Higgins' I^2^ test*. The publication bias was examined via funnel plot and *Hegger's test*.

**Results:**

Our meta-analysis was conducted based on 254 diagnosed with Autism Spectrum Disease cases and 161 healthy cases from 4 studies. Compared to the healthy cases, the odds of presence of Candida albicans (OR=7.21; 95% CI: 3.75-13.85; p<0.001) were higher in those diagnosed with autism spectrum disorder.

**Conclusion:**

This study as a whole showed that children diagnosed with autism spectrum disorder have higher frequency of the presence of the fungus Candida albicans. Therefore, Candida albicans may be an etiological factor for the autistic behavior in children.

## Introduction

Autism spectrum disorder (ASD) is a group of neurologic disorders identified by repetitive movements, difficulties in socialization and interaction, narrow interests and language development deficiencies.

In the past, ASD prevalence was approximately 4 to 5 in 10000 children and it was considered as a rare disorder. However, a dramatic increase has been observed in the number of ASD cases such that the prevalence of autism has advanced to 1 in 160 people in 2019 and it becomes a common disorder now.^1^

Due to the high increase in the rate of autism, it has gained great importance to determine the etiology of ASD. While some scientists attribute the etiology of autism to genetic factors, there are some studies that indicate the effects of the other factors such as intestinal flora, antibiotic use during pregnancy and exposure to air pollution on autism.^3^

In this study, we were interested in the intestinal flora from the causes of ASD and aimed to explore the effect of Candida albicans on ASD, which is a commensal fungus disrupting the intestinal flora. It is mostly seen in women and 75% of women encounter this fungus at least once in their lifetime.^4^ Although it is often a benign member of the skin and mucous flora, it has polymorphic abilities and it can transform into a pathogenic and invasive fungal form in some cases.^5^ Emam et al.^6^ revealed that an intense development of Candida albicans was seen in the stool culture of autistic children. Iovene et al.^7^ showed that Candida albicans growth was measured higher in children with ASD via a simple cultural approach. Similarly, Hughes and Ashwood 8 indicated that children with ASD had a higher percentage of positivity for the presence of Candida albicans than those in the healthy control group. Lastly, Koceski and Trajkovski^9^ pointed out that the fungus Candida albicans was more common in people with ASD compared to the control group in their study. Therefore, Candida albicans was thought to have a great effect on the intestinal flora, consequently on autism. The purpose of our study was to assess the role of Candida albicans as a risk factor to cause autism behavior.

## Material and methods

### Study selection and eligibility criteria

Meta-analysis was carried out according to the Preferred Reporting Items for Systematic Reviews and Meta-Analyses (PRISMA) guidelines. We searched Scopus, PubMed, Web of Science and PsycINFO for articles up to December, 2021. Literature searches conducted based on title, abstract, and keywords. The terms “Autism” AND “Candida” were used for searching the publications. Any language or country restriction was not made. Two authors examined the studies' titles and abstracts independently for inclusion eligibility.

The following criteria were handled in the selection of studies:

- The studies involving children diagnosed with ASD were included.
- Children' outcomes were selected as Candida albicans (positive) and Candida albicans (negative).
- Studies examining the associations between ASD and presence of Candida albicans were included.
- Studies reporting count and percentage for binary variables were included.

### Statistical analysis

STATA version 15 and IBM SPSS 20.0 (IBM Corp., Armonk, NY, USA) were used for the statistical analyses. Presence of Candida Albicans was summarized as counts (percentages). For each study, Chi-square test was employed in the comparisons of presence of Candida albicans between autism and control groups. Odds ratios (OR) with 95% confidence intervals (CI) were reported using fixed-effect and random-effect meta-analysis with *“metan”* command. Higgins' I^2^ and Chi-square tests were conducted to assess the heterogeneity of the studies. In the presence of heterogeneity, random-effect meta-analysis was performed. The publication bias was examined via funnel plot and Hegger's test. A p-value<0.05 was accepted for significance.

## Results

### Identified studies

**Figure 1:**
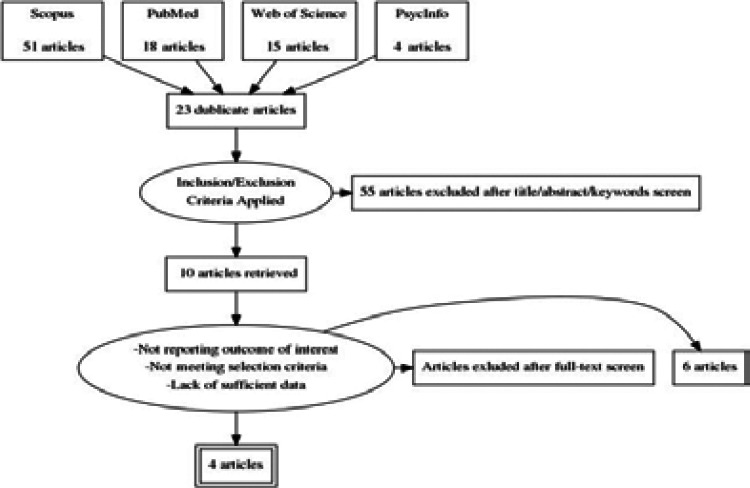
PRISMA flow diagram for selection of studies

Database searching identified totally 88 studies (51 studies in Scopus, 18 studies in PubMed, 15 studies in Web of Science and 4 studies in PsycINFO). The 23 duplicate studies were removed. The 55 studies were excluded through reviewing the titles and abstracts. The remaining 13 studies were applied exclusion criteria such as not reporting outcome of interest, not meeting selection criteria and lack of sufficient data and 6 of them were removed. Therefore, 4 studies were retained, which fulfilled inclusion criteria (Figure 1). Characteristics of these studies were given in Table 1.

**Table 1:** Study characteristics

Studies	Countries	Exclusion criteria	Sample sizes ASD-Control	Age Mean±SD / Median [IQR]	Male n (%)
Emam (08, 2012)	Egypt	1. Children under antifungal treatment. 2. Children with abnormal routine laboratory investigations. 3. Children under immunosuppressi ve or cytotoxic drugs.	83-25	ASD: 47.44±7.41; months Control: 44.19±6.25; months	ASD: 68 (81.9) Control: 15 (60)

Iovene (08, 2017)	Italy	1. Autism secondary to genetic syndromes 2. Rett syndrome 3. Childhood disintegrative disorder 4. Epilepsy 5. Neurological syndromes 6. BMI<25th or>85th percentile 7. Concomitant condition of known CD 8. DMT1	47-33	ASD: 6.0±2.8; years Control: 7.3±3.1; years	ASD: 40 (85.1) Control: 24 (72.7)

Hughes (11, 2017)	United States	1. IBD or other GI pathology. 2. Participants having recent evidence of a GI infection. 3. Participants taking medication altering GI function. 4. Participants with seizure, genetic and chronic diseases or infections.	52-28	ASD: 7.42 [5.17-9.42]; years Control: 6.5 [5.58-8.33]; years	ASD: 44 (84.6) Control: 25 (89.3)

Koceski (05, 2021)	Macedonia	-	72-75	ASD: 10.53±4.07; years Control: 10.37±3.68; years	116 (79.0)

*CD: Celiac Disease, DMT1: Diabetes Mellitus Type 1, IBD: Inflammatory Bowel Disease, GI: Gastrointestinal*

### Meta-analysis results

**Figure 2:**
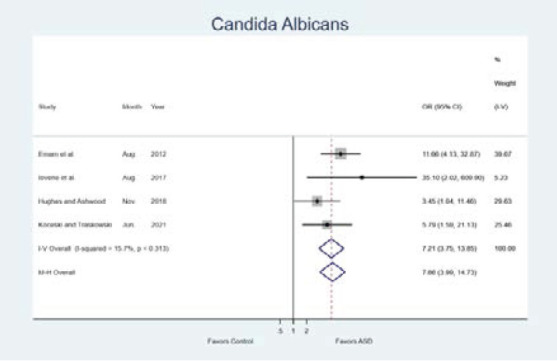
Forest plots for the presence of Candida albicans

The comparisons of presence of Candida albicans between autism and control groups were given in Table 2. All studies taken to the meta-analysis reported significant difference between ASD and control groups for the presence of Candida albicans. However, there were great differences between these studies in terms of the obtained odds ratios. Meta-analysis results were also given via forest graphs in Figure 2. Figure 2 showed that the odds of presence of Candida albicans (OR=7.21; 95% CI: 3.75-13.85; p<0.001) were statistically greater in ASD group compared to the control group.

**Table 2:** Comparisons of ASD and control groups with respect to Candida presence given by each study

Studies	Month, Year	Control, n (%)	ASD, n (%)	*p*
Emam et al.^6^	08, 2012	n=25 7 (28.0)	n=83 68 (81.9)	**<0.001**

Iovene et al.^7^	08, 2017	n=33 0 (0)	n=47 16 (34.0)	**0.001**

Hughes and Ashwood^8^	10, 2018	n=28 4 (14.3)	n=52 19 (36.5)	**0.041**

Koceski and Traskowski^9^	06, 2021	n=75 3 (4.0)	n=72 14 (19.4)	**0.008**

*Bold faces indicate statistically significant values*.

### Publication bias

One of the important problems in conducting a meta-analysis is the publication bias. It may occur frequently when the studies showing significant differences are more likely to be published or studies are preferentially published in English language journals and higher impact journals. Since the publication bias can lead to inflated estimates of efficacy, the presence of publication bias should be explored. There are graphical and statistical methods to identify the publication bias. The main graphical method is the funnel plot. In our study, funnel plot was used firstly to examine the publication bias. The funnel plot gives a visual representation of the potential bias and its interpretation is subjective. Therefore, we also used Egger's test which is one of the most commonly used tests to assess the publication bias.

**Figure 3:**
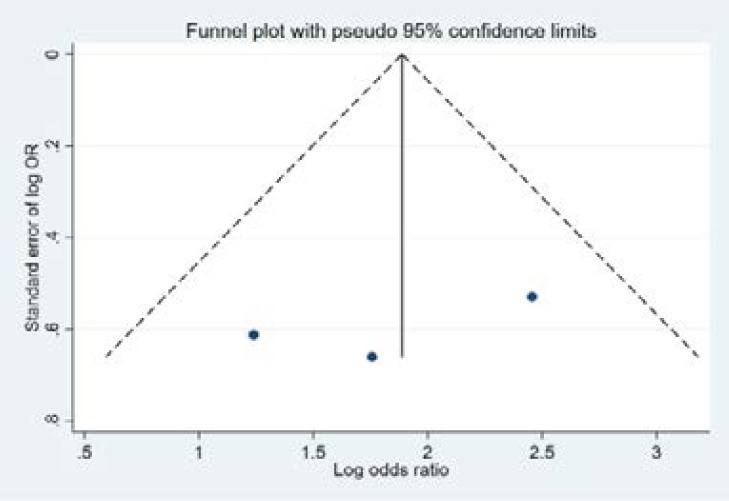
Funnel plots; presence of Candida Albicans

For Candida albicans, the obtained funnel plot was provided in Figure 3. Since it was qualitatively symmetrical and inverted funnel, it could be considered that there was no publication bias. Egger's test also showed no publication bias (t= -1.13; p=0.462).

### Study heterogeneity

In a meta-analysis study, which gives a summary result combining several studies, the homogeneity of the combined studies is a very important assumption. Therefore, we tested this assumption using *Chi-square test* and quantified it by *I*^*2*^ statistic. Heterogeneity below 25% was taken as none, 25%-50% as low, 50%-75% as moderate and above 75% as high. No heterogeneity was obtained for Candida albicans (*X*^2^=3.56, p=0.313; *I*^2^=15.7%).

## Discussion

Although the exact etiology of autism is still unknown, it is considered that autism results from a combination of genetic, environmental, neurological and immunological factors. Genetic factors are dominant in the development of autism; however, recent studies claim that gut microbiota influence the behavior of autism and the intestinal flora became one of the important topics in ASD researchs.^10^,^11^ Fungi are important part of the human intestinal flora and may have an harmful impact on human health.^12^ Especially Candida albicans fungus is thought to lead to autistic behaviors by decreasing the minerals and carbohydrates absorption and increasing the toxin levels.^13^-^15^

In this study, whether Candida albicans fungus can be a risk factor for autism was examined with a meta-analysis study. The meta-analysis study was conducted based on 4 studies investigating the relationship between the autism and the presence of Candida albicans. A total of 415 people from Egypt, Italy, Macedonia and USA were involved in these studies. While 254 of them were diagnosed with autism, 161 were not.

Although all studies taken to the meta-analysis showed that a statistically significant relation exists between the Candida albicans and autism, there were great differences between these studies in terms of the obtained odds ratios. The odds ratios were ranging from 3.45 to 35.10. It was seen that the presence of Candida albicans increased the risk of autism approximately 12 times in the study of Emam et al.^6^, 35 times in the study of Iovene et al.^7^, 3.5 times in the study of Hughes and Ashwood^8^ and 6 times in the study of Koceski and Trajkovski^9^ (Figure 2). Therefore, we conducted a meta-analysis to provide more consistent information about the relation between the presence of Candida albicans and autism. Our meta-analysis results revealed that the presence of albicans increases the risk of autism about 7 times (OR=7.21; 95% CI: 3.75-13.85; p<0.001).

The strength of our study is lies in the following points:

- As far as the authors' knowledge, this is the first meta-analysis conducted on this subject.
- There was no heterogeneity and no publication bias.
- The database Scopus, PubMed, Web of Science and PsycINFO were searched independently by two authors and the predetermined eligibility criteria were used in the publication selection to ensure the quality of the study. In conclusion, this study as a whole showed that children diagnosed with ASD have higher frequency of the presence of the fungus Candida albicans. Therefore, Candida albicans may be an etiological factor for the autistic behavior in children.
